# Investigating the Impact of Antioxidant Supplementation on Male Infertility: A Scoping Review

**DOI:** 10.3390/jcm15020497

**Published:** 2026-01-08

**Authors:** Emmanouil Andreou, Charalampos Karachalios, Paraskevas Perros, Ilias Liapis, Georgia Koutsogeorgopoulou, Eftichia Katagi, Marios-Nektarios Filis, Alexandros Nakis, Vasileios Tzikoulis, Athanasios Chionis, Konstantinos Daglas, Angeliki Papadimitriou, Christos-Konstantinos Michalopoulos, Antonios Lagadas

**Affiliations:** 1 Faculty of Health Sciences, School of Medicine, Aristotle University of Thessaloniki, 54124 Thessaloniki, Greece; emandreouu@gmail.com (E.A.); koutsogeorgopoulouz@yahoo.gr (G.K.); bill1996tziko@gmail.com (V.T.); ckmichalopoulos@gmail.com (C.-K.M.); 2 Gynecology Clinic, General Hospital of Athens “LAIKO”, 11527 Athens, Greece; paris_per@yahoo.gr (P.P.); eftichiakatagi68@gmail.com (E.K.); mariosn.filis@gmail.com (M.-N.F.); alexandrosnakis@gmail.com (A.N.); ath.chionis@yahoo.gr (A.C.); daglask@gmail.com (K.D.); apapadimitriou@otenet.gr (A.P.); alagadas@gmail.com (A.L.); 3 Birmingham Women’s Hospital, Birmingham B15 2TG, West Midlands, UK; liapisilias7@hotmail.com

**Keywords:** antioxidant, oxidative stress, male infertility, sperm DNA oxidation, semen analysis

## Abstract

Infertility affects thousands of couples internationally, leaving a profound effect on their families and communities. According to the World Health Organization (WHO), approximately one out of six individuals of reproductive age worldwide experiences infertility in their life span. Approximately 35% of infertile couples are affected by male factor infertility, in which semen analysis is the gold standard diagnostic procedure. Oxidative stress (OS) is considered to play a pivotal role in the pathogenesis of male infertility. A thorough literature search was conducted in PubMed/MEDLINE, Scopus and Google Scholar databases, using MeSH terms and free-text keywords, to retrieve eligible articles published in the last decade, focusing on the potential beneficial role of oral antioxidants in male infertility. Antioxidant supplementation appears to improve the majority of sperm parameters. Therefore, antioxidant therapy is emerging as a promising aid in addressing male infertility. The purpose of this comprehensive literature review is to evaluate the significance of antioxidant supplementation in improving sperm parameters. Most of the included randomized controlled trials demonstrated the positive effects of oral antioxidants in various parameters, such as sperm concentration, total sperm count, motility and progressive motility. Consequently, pregnancy outcomes were evaluated, and increased pregnancy rates were reported in the majority of the included studies.

## 1. Introduction

Infertility is defined as the inability to conceive after a year of unprotected intercourse sessions of reasonable frequency. It can be subdivided into primary infertility, in cases where the couple has never conceived in the past, and secondary infertility, referring to couples who have at least one previous full-term pregnancy [1]. In cases where the female partner is older than 35 years old, infertility is defined as failure to conceive within six months of unprotected intercourse. Therefore, the delay in extensive evaluation and treatment is not reasonable [2]. Conversely, fecundability refers to the likelihood of achieving conception within a single menstrual cycle [3]. Data from large-scale population studies indicate that the monthly probability of conception ranges between 20% and 25%. Among couples actively attempting to conceive, over 85% are expected to achieve pregnancy within one year [4,5]. In approximately 35% of couples with infertility, both male and female factors are concurrently identified, whereas in about 10% of cases, male factor infertility is the sole identifiable cause [6,7].

Several factors result in substantial disparities in access to infertility care. Diagnosis of conditions in men may be delayed or missed, due to social misconceptions that “hold” the female “primarily responsible” for conception and childbearing [8]. The diagnostic approach of male infertility is focused on detecting the small percentage of causes that can be treated, so as to achieve fertilization. The initial evaluation is focused on determining which couples with male infertility factors might benefit from assisted reproductive technologies (ART) [9,10].

The evaluation of infertile men typically comprises three fundamental components: a thorough medical history, physical examination, and semen analysis. Depending on the clinical presentation, additional diagnostic assessments may be warranted, including endocrine evaluation, imaging of the accessory sex glands and reproductive ducts, and, lastly, genetic tests. A detailed analysis of the history and physical examination falls beyond the scope of the present review. Conversely, semen analysis is the fundamental laboratory diagnostic tool for evaluating the male partner of an infertile couple and warrants further clarification [11,12]. The semen analysis should be performed using standardized methodology, preferably as described in the (WHO) Laboratory Manual for the Examination and Processing of Human Semen. In case of an abnormal semen analysis, one or two additional analyses are recommended to confirm the initial findings. Consequently, a sperm concentration of 15 million spermatozoa/mL is considered the lower threshold of normal [13]. The generally accepted parameters of semen analysis are presented below [14]:

- pH: 7.2–7.9
- Volume: 1.4 mL (95% CI 1.3–1.5 mL)
- Total sperm number: 39 million spermatozoa per ejaculate (95% CI 35–40 million)
- Sperm number/mL: >15 million spermatozoa per ejaculate
- Morphology: 4% normal forms (95% CI 3.9–4%)
- Vitality: 54% live (40–43%)
- Progressive motility: 30% motile (95% CI 29–31%)
- White blood cells: <1 × 10^6^/mL
- Round cells: <5 × 10^6^/mL

In general, infertility can be attributed to the female partner in one third of cases, the male partner in one third of cases, and both partners in the remaining one third [15]. This approximation emphasizes the value of assessing both partners. While many infertile men present oligozoospermia, namely a reduced sperm count relative to reference values, or azoospermia, which is the complete absence of sperm cells in the ejaculate, some infertile men will have normal sperm counts [10]. More than 80% of infertile men will demonstrate a reduced sperm concentration and poor sperm quality, which is defined as a decrease in sperm motility, namely “asthenozoospermia” [16]. Furthermore, another sperm disorder refers to the increased presence of abnormal morphologically spermatozoa, a state called “teratozoospermia”. A few percent of men who are unable to conceive will have normal sperm quantities, but low sperm quality. Another small percentage of infertile men will have normal sperm concentrations, as well as normal motility and morphology without any abnormalities [10,17].

An abnormal semen analysis should be further investigated with more specialized testing, including scrotal ultrasound, epididymal biopsy, semen culture, karyotype, screening for cystic fibrosis and testing for sperm antibodies. The causes of male infertility can be divided into four main areas [18]:

- Endocrine and systemic disorders with hypogonadotropic hypogonadism
- Primary testicular defects in spermatogenesis
- Sperm transport disorders
- Idiopathic male infertility

Oxidative stress (OS) is one of the causes most implicated in the pathogenetic mechanism of infertility [19,20]. In the female reproductive system, a finely regulated balance between reactive oxygen species (ROS) and antioxidants is essential for maintaining redox homeostasis [21]. Although the precise mechanisms by which OS impairs sperm quality remain unclear, it is well established that reduced intracellular adenosine triphosphate (ATP) levels, inadequate axonemal phosphorylation, and lipid peroxidation of the sperm membrane contribute to diminished motility and functional impairment, ultimately reducing the sperm’s capacity to fertilize the oocyte [22,23]. Antioxidants could protect against the damaging effects of leukocyte-derived ROS on sperm movement and may be of clinical value in assisted conception procedures [24].

The main objective of the present comprehensive literature review is to summarize the current evidence on whether oral antioxidant supplementation can assist infertile men in improving their sperm parameters and, thus, maximize their potential of achieving a clinical pregnancy outcome.

## 2. Materials and Methods

This is a scoping review of the literature aiming to summarize evidence from the last decade regarding the potential beneficial role of oral antioxidant supplementation in improving sperm parameters in infertile men, thereby leading to higher clinical pregnancy rates.

### 2.1. Searching Strategy

First, we conducted a systematic search of the literature to identify studies analyzing the role of antioxidant therapy in male infertility. PubMed/MEDLINE, Scopus, and Google Scholar databases were searched up to 31 March 2025, using MeSH terms and free-text keywords. The search strategy involved combining the following terms with the Boolean operators “AND” and “OR”: “antioxidant”, “oxidative stress”, “male infertility”, “spermatozoa”, “semen”, “male subfertility”, “sperm DNA oxidation”, “asthenozoospermia”, “asthenospermia”, “oligospermia”, and “oligoasthenospermia”.

#### Eligibility Criteria

The literature search covered the period from 1 January 2015 to 31 March 2025, focusing on fully published, full-text English journal articles involving human adult males over 19 years old. Only prospective and retrospective randomized controlled trials (RCTs) were considered eligible. The focus on RCTs exclusively aims to derive more reliable and causally interpretable conclusions regarding the intervention’s effects. Additionally, only RCTs were included in this scoping review, as the randomized design minimizes selection bias and confounding. Lastly, we performed manual searches of references listed in initially identified articles. Cross-referencing references of included studies was also conducted to identify further eligible studies for inclusion in this review.

### 2.2. Exclusion Criteria

Studies would be excluded in cases where they lacked information about changes in sperm parameters before and after oral antioxidant supplementation in infertile men. Furthermore, non-randomized controlled trials were excluded from this review, as specified in the predefined inclusion criteria. Animal studies, systematic reviews, meta-analyses, abstract-only publications, protocols, video reports, and non-English articles were also excluded.

### 2.3. Study Selection Process

All studies identified from the search strategy were imported into a reference management software (Mendeley Reference Manager, Version 2.138.0) for the elimination of duplicate records and further assessment. Titles and abstracts of all the retrieved articles were assessed by two independent reviewers (E. A. and P. P.) based on their full-text manuscripts, while articles irrelevant to the objective of the present review were excluded. If a study was selected by only one reviewer, the decision for inclusion was taken by a third reviewer (A. L.). Considering the heterogeneity of the article types, we decided to represent the results as a scoping review.

## 3. Results

### Study Selection

**Figure 1:**
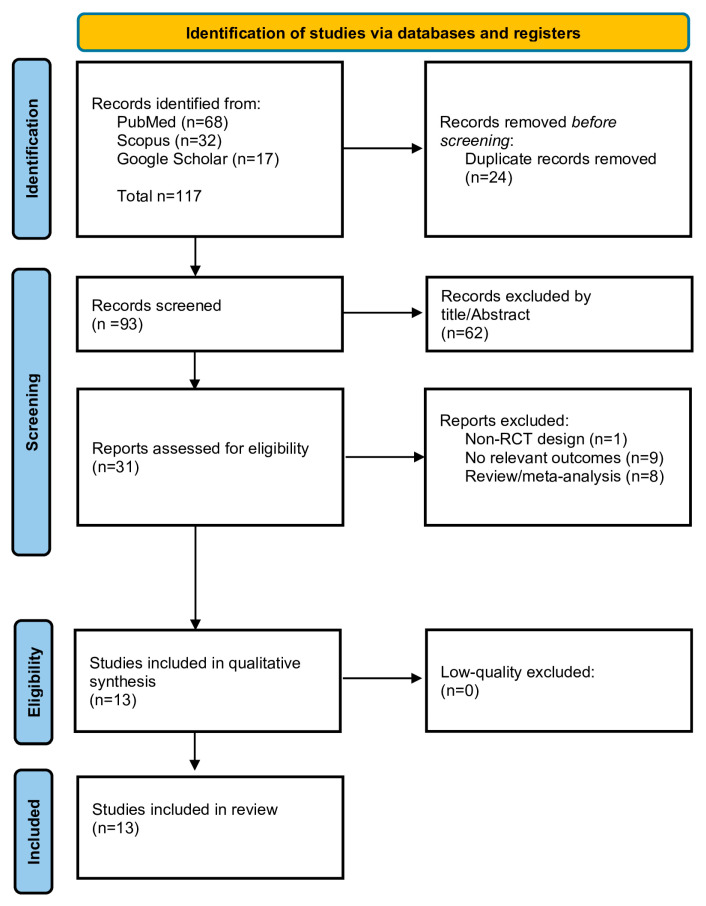
Flowchart of study selection.

Electronic searches and additional hand-searching retrieved 117 articles. After the exclusion of 24 duplicate records, 93 records were screened by title/abstract. Afterwards, 58 articles were excluded and 35 studies were assessed for eligibility based on their full-text manuscript. After thorough evaluation, 14 studies met the inclusion criteria and were selected for data extraction, in particular, four double-blind randomized controlled trials (DB-RCTs), eight randomized controlled trials (RCTs), one double-blind, placebo-controlled trial (DBPC), and one clinical trial. The flow diagram in Figure 1 schematically presents the stages of article selection. An overview of the included articles is presented in Table 1. All studies were performed in men with idiopathic male infertility and were published between 2015 and 2024. A PRISMA checklist specifically designed for scoping reviews (PRISMA-ScR) has been filled out and is provided as supplementary material (Table S1). In addition, a risk-of-bias assessment table, designed according to the revised Cochrane tool to assess risk of bias in randomized trials (RoB 2), is also provided as a supplementary file (Table S2).

Recently, Busetto et al. [25] assessed the role of a variety of antioxidant supplementation in the management of oligoasthenoteratozoospermia and varicocele. The researchers included a total of 47 men with varicocele and 47 without varicocele. The volunteers received a supplementation formulation of 1000 mg L-carnitine, 725 mg fumarate, 500 mg acetyl-L-carnitine, 1000 mg fructose, 20 mg Coenzyme Q10 (CoQ10), 90 mg vitamin C, 10 mg zinc, 200 μg folic acid, and 1.5 μg vitamin B12 or a placebo daily for 6 months. Antioxidants had increased sperm concentration and total sperm count in the intervention group. The supplementation also had a positive effect on sperm motility, but no effect on sperm morphology.

Similarly, Helli et al. [26] assessed the effects of probiotics on sperm parameters, oxidative index, inflammatory factors and sex hormones in infertile men. The researchers included 25 infertile men in the control group and 25 infertile men in the intervention group. The latter were administered a combination of 500 mg probiotic capsules—Lactobacillus cases, Lactobacillus rhamnosus, Lactobacillus bulgaricus, Lactobacillus acidophilus, Bifidobacterium breve, Bifidobacterium longum and Streptococcus thermophiles—for 10 weeks. At the end of the study, probiotics produced significant changes in ejaculate volume, total sperm count, sperm concentration and sperm total motility. No significant changes were found in sperm morphology.

Maghsoumi-Norouzabad et al. [27] associated vitamin D3 (VD3) supplementation with spermogram and oxidative stress biomarkers. They conducted a randomized, triple blind, placebo-controlled clinical trial that included 86 asthenozoospermic infertile men with serum 25-hydroxy vitamin D3 < 30 ng/mL. VD3 supplementation of 4000 IU for 3 months was given in the intervention group. No significant effects on semen volume, sperm count, and sperm morphology were detected. However, authors observed increased seminal and serum total antioxidant capacity (TAC), total sperm motility and progressive sperm motility. Additionally, they have shown a decrease in seminal and serum malondialdehyde (MDA).

The first study in the present review to evaluate the role of combined effect of lifestyle intervention and antioxidant therapy on oxidative stress in patients undergoing in vitro fertilization (IVF) was conducted by Humaidan et al. [28] In this pilot study, researchers enrolled 31 infertile males with a history of failed IVF/intracytoplasmic sperm injection (ICSI) and 10 healthy male volunteers as controls. All males with a DNA Fragmentation Index (DFI) > 15% received a combination of a daily supply of CoQ10 100 mg, one multivitamin (Apovit), and 1 g Omega-3. After 3 months of lifestyle modifications and antioxidant therapy, a new semen sample was obtained. It was then demonstrated that antioxidant supplementation and lifestyle modifications resulted in significantly decreased DFI values, but no significant effect was seen on sperm concentration or total motile sperm count.

In their randomized control trial, Knudson et al. [29] investigated the association of vitamin E, selenium, and zinc in the management of plasma antioxidant levels of infertile men. They were subsequently randomized to a placebo or an antioxidant formulation containing 500 mg of vitamin C, 400 mg of vitamin E, 0.2 mg of selenium, 1000 μg of folic acid, 1000 mg of L-carnitine, 20 mg of zinc, 2000 IU of vitamin D and 10 mg of lycopene for up to 6 months. According to their findings, no correlation was detected with semen parameters or clinical outcomes in couples with infertility issues.

Haidari et al. [24] sought to determine whether lipoic acid (LA) supplementation was correlated with gene expression and activity of glutathione S-transferase (GST) enzyme in infertile men. This randomized, triple-blind, placebo-controlled trial was conducted on 23 infertile men receiving 600 mg LA and 21 infertile men receiving a placebo for 12 weeks. At the end of the study, testosterone, GST gene, total sperm count, sperm concentration and sperm motility were increased in the tested group.

Alahmar et al. [30] conducted a randomized controlled trial trying to correlate the impact of CoQ10 and selenium on seminal parameters. The selected patients were randomized and separated into 2 Groups. Group 1 received 200 mg of oral CoQ10 as a single daily dose, whilst Group 2 received 200 μg of selenium orally for 3 months. Similar results were reproduced with the increase in sperm parameters in the CoQ10 group. No significant changes were found in the selenium group.

Aghajani et al. [31] studied the effect of Ceratonia siliqua L. syrup (carob) and vitamin E on sperm parameters, oxidative stress and sex hormones in infertile men. The researchers recruited 30 males with oligozoospermia, asthenospermia, and teratospermia, who were given 7.5 mL of carob syrup, and 30 infertile men, acting as an active group, who were supplied with 100 mg of vitamin E twice a day for three months. The results of this study showed that treatment with carob for 90 days significantly improved semen parameters, and it was also found that in the same group, there was an increase in TAC and a reduction in MDA.

Similar results were reproduced by Eslamian et al. [32]. In their randomized controlled trial, asthenozoospermic men were randomly assigned to 1 of 4 groups according to age and sperm concentration. Participants were supplied daily with 465 mg docosahexaenoic acid (DHA) plus 600 IU vitamin E (DE), 465 mg DHA plus placebo (DP), 600 IU vitamin E plus placebo (EP), or both placebo (PP) for 12 weeks. Conversely, total sperm count, concentration, motility, and progressive motility were significantly increased by supplementation with DE, EP, and DP compared with PP (*p* < 0.05). The most significant effect was seen in the DE group, followed by the EP and DP groups, on all parameters. MDA decreased significantly in the DE and EP groups and was considerably less in these groups than in the PP and DP groups (*p* < 0.001).

Nouri et al. [33] in their retrospective analysis studied 36 oligospermic infertile men who were divided into two groups. The first group (n = 17) had taken 25 mg of lycopene for 12 weeks, and the other (n = 19) was administered a placebo. The researchers found that there was a statistically significant increase in the lycopene group, as far as total sperm count, sperm concentration and sperm motility are concerned.

Stenqvist et al. [34] in a double-blind, randomized study evaluated the effects of combined antioxidant treatment in 77 men from infertile couples, with normal testosterone, luteinizing hormone (LH) and follicle-stimulating hormone (FSH) levels and DFI ≥ 25%. The treatment group (n = 37) received a combination of vitamins (vitamin C 30 mg, vitamin E 5 mg and vitamin B12 0.5 mg), antioxidants (L-carnitine 750 mg, coenzyme Q10 10 mg and folic acid 100 mg) and trace elements (zinc 5 mg and selenium 25 mg) with maltodextrin, calcium carbonate, citric acid, steviol glycoside, flavors, beta-carotene and silicon dioxide) for 6 months. The authors found a borderline statistically significant higher sperm concentration, but no other improvements in sperm parameters, among the two groups.

Alsalman et al. [35] studied 60 infertile male partners from couples who had referred to the infertility clinic of Babylon Hospital of Maternity between July 2011 and July 2012. The study group was administered 220 mg of zinc twice per day for three months. The authors compared their semen analysis before and after the zinc administration and with a male fertile group (n = 60). They concluded that the quantitative values for thiol oxidoreductive index and thiol-related enzymes may provide a useful means to qualitatively express the oxidant/antioxidant balance in clinical and epidemiologic studies. Zinc supplementation may restore the thiol oxidoreductive index and thiol-related enzyme activities to normal ranges in seminal plasma and in spermatozoa of asthenozoospermic subjects.

In a double-blind randomized control study, Alizadeh et al. 2017 [36] compared 28 infertile men to a similar quantitative control group. In the infertile group, participants were administered 80 mg of curcumin nanomicelle for 10 weeks. The authors observed that in the infertile group, total sperm count, sperm concentration, and sperm motility were statistically increased and were comparable to the placebo group. In addition, they noticed statistically increased plasma levels of TAC, MDA, C-reactive protein, and tumor necrosis factor in comparison to the control group.

Haghighian et al. [37] conducted a double-blind randomized controlled study that evaluated 44 subfertile men. They divided them into two groups. In the first group (n = 23), men were given 600 mg alpha-lipoic acid (ALA), and in the second group (n = 21), they were given a placebo for 12 weeks. ALA supplementation, compared with placebo, significantly increased sperm concentration, sperm count, and sperm total motility, whereas parameters like ejaculate volume and morphology were not significantly different.

## 4. Discussion

Our review aimed to evaluate the effects of antioxidant supplementation on male infertility. Evidence from the 14 included studies suggests that probiotic supplementation may enhance various semen parameters, including motility, sperm concentration, morphology, semen volume, and total sperm count. The potential therapeutic effects of antioxidant supplementation have been explored across various medical fields.

### 4.1. Sperm Parameters

Almost all articles included in the present review reported improvement in sperm parameters. Among all studies reviewed, Knudtson et al. [29] was the only one to report findings that contradicted the remaining studies. No association was found between baseline levels of selenium, zinc, or a-tocopherol and semen parameters, including sperm concentration, motility, total motile count, or DNA fragmentation. Similarly, after 3 months of treatment, antioxidant levels remained uncorrelated with antioxidant levels and semen parameters or DNA fragmentation. Busetto et al. [25] concluded that there was a statistically significant increase in sperm concentration and total count in the overall patient group undergoing antioxidant therapy, compared to patients receiving the placebo (*p* < 0.05). The antioxidant treatment had a positive effect on sperm motility, although it did not reach statistical significance (*p* > 0.005). Lastly, there was no significant difference in sperm morphology. Helli et al. [26] found that the intervention group had statistically significant changes in ejaculate volume, total sperm count, sperm concentration, and sperm total motility. In the study by Humaidan et al. [27], participants underwent a three-month period of oral antioxidant supplementation and a lifetime intervention with reduced DFI. Still, no differences in sperm concentration and total motile sperm count were observed. Alahmar et al. [30] compared the impact of CoQ10 and selenium supplementation in infertile men with idiopathic oligoasthenoteratospermia. Following selenium and CoQ10 therapy, there was a significant improvement in sperm concentration in the CoQ10 group (*p* < 0.01), but not in the selenium group (*p* > 0.05). An increase in progressive motility was observed in both groups, but higher in the CoQ10-treated subjects than in the selenium group (*p* < 0.01 and *p* < 0.05, respectively). Total motility was also significantly increased with both treatments, but stronger with CoQ10 than selenium (*p* <0.01 and *p* < 0.05, respectively). There were no significant changes in morphology observed in either of the groups. Aghajani et al. [31] concluded that there were no significant differences between the means of pretreatment volume between the two groups. A significant improvement in semen count and motility was found in the carob group. Moreover, a significant reduction in sperm morphology was also found. Eslamian et al. [32] presented the results from the analysis of semen parameters. They showed a significant increase in total sperm count, concentration, motility, and progressive motility with supplementation.

Our findings align with the systematic review by Licia Cristina Silver de Lima Oliveira et al. [38], which observed significant improvements across all sperm parameters, demonstrating marked enhancement in motility. They showed a potential therapeutic alternative for infertile men with the supply of probiotic supplementation. Both the current review and the double blind, randomized control trial of Helli et al. [26] reached similar conclusions, highlighting the role of probiotic supplementation in the therapeutic approach of the infertile men. Similarly, the meta-analysis of Barbonetti et al. [21] identified a specific group of antioxidant compounds potentially valuable for idiopathic forms of infertility. Di Tucci et al. [39] published a systematic review to associate the role of alpha-lipoic acid in female and male infertility. The results showed that ALA can improve sperm quality. Similar results revealed the significant role of carnitines in the improvement of sperm parameters. Lastly, Cannarella et al. [40] investigated the potential role of Serenoa repens, selenium and lycopene. They drew similar conclusions to those reported in our study. Their review suggested the importance of the combined administration of these supplements in improving sperm quality.

### 4.2. Hormones

Three studies observed the outcome of hormonal status upon conclusion of the intervention [24,26,28,31]. Helli et al. [26] observed no significant difference in sex hormones between the two intervention and placebo groups. The probiotic supplementation after 10 weeks could increase testosterone levels and decrease FSH, LH and prolactin (PRL). However, these differences did not reach statistical significance. Haidari et al. found similar results when comparing the supplemented group to the placebo, identical to those of Helli et al. [26] after a 12-week intervention period (*p* > 0.05). After 12 weeks of treatment, serum testosterone increased 12.28% from baseline. Aghajani et al. [31] compared the effects of C. siliqua (carob) on semen parameters, vitamin E on semen parameters, oxidative stress, and pregnancy rate. In addition, the level of testosterone was improved significantly in the carob group (*p* > 0.05). LH levels were decreased in both groups, but more significantly in the Vitamin E group (*p* = 0.007).

### 4.3. Pregnancy Rate

Three studies evaluated the pregnancy rate after the duration of antioxidant therapy [25,29,31]. While pregnancy was not the primary outcome, Busetto et al. [25] observed a total of 12 pregnancies during the trial, with ten pregnancies occurring in the intervention group. Additionally, two pregnancies were observed in the placebo group. Notably, one spontaneous abortion was reported in the placebo group as well. This observation aligns with the noted improvements in semen parameters, highlighting the potential clinical relevance of antioxidant supplementations. Aghajani et al. [31] have noticed a 23% pregnancy rate in the carob group (5 spontaneous pregnancies and 2 through intrauterine insemination, IUI). In the Vitamin E group, the pregnancy rate was 13% (1 natural conception and 3 by IUI). It should be pointed out that no significant difference between groups was observed. One intrauterine fetal death and one preterm labor were seen in the control group, and one low birth weight neonate and two preterm labors were reported in the Vitamin E group. Knudtson et al. [29] ended up with no correlation with pregnancy or live birth among all couples who enrolled in the trial, regardless of treatment allocation. No difference was found between the placebo and intervention groups among those who conceived and had a live birth and those who did not conceive or have a live birth.

The findings of our study are in agreement with those of Wiep de Ligny et al. [41], who also demonstrated a notable increase in clinical pregnancy rates and live birth rates.

## 5. Conclusions and Future Directions

This review underscores the potential therapeutic value of antioxidant supplementation for addressing male infertility. The majority of the clinical trials reported significant improvements in key semen parameters. Although the effects on sperm morphology and hormonal profiles were less consistent, antioxidant intervention generally demonstrated positive results, especially in instances of idiopathic oligoasthenoteratozoospermia. Notably, several studies reported a higher incidence of spontaneous pregnancies, implying a possible correlation between antioxidant therapy and enhanced clinical fertility outcomes. Despite these results, we found heterogeneity among studies, including sample sizes, antioxidant compositions, and treatment durations. Furthermore, the absence of long-term follow-up data underscores the need for more large-scale randomized controlled trials. Nevertheless, the differences in the reviewed guidelines, although limited, highlight the need for large multicenter, well-designed RCTs to explore the entire spectrum of antioxidants’ potential therapeutic usage and improve the chances of infertile couples, with a focus on the male factor. In summary, more homogeneous studies are warranted to clarify the role of antioxidants in the treatment of male infertility.

## Figures and Tables

**Table 1:** Overview of Published Studies Assessing Supplementation on Male Infertility.

Authors	Duration	Design of Study	Sample Size		Supplementation	Outcome
			Intervention Group	Control Group		
Busetto et al. 2024 [25]	2014–2015	DBPC-RCT	47	47	1 g L-carnitine, 725 mg fumarate, 500 mg acetyl-carnitine, 1 g fructose, 20 mg CoQ10, 90 mg VitC, 10 mg zinc, 200 μg folic acid, 1.5 μg Vit B12 or placebo for 6 months	↑ sperm concentration ↑ total sperm count Positive effect on sperm motility no effect on sperm morphology
Helli et al. 2022 [26]	2018–2019	DB-RCT	25	25	Combination of probiotics combination capsules or placebo for 10 months	↑ ejaculate volume ↑ total sperm count ↑ sperm concentration ↑ sperm total motility ↑ live sperm
Maghsoumi-Norouzabad et al. 2022 [27]	2018–2021	RCT	43	43	Vitamin D3 or placebo for 3 months	↑ Seminal and serum TAC, ↑ total sperm motility, ↑ progressive sperm motility, ↓ seminal and serum MDA
Humaidan et al. 2022 [28]	2018–2020	Clinical Trial	31	10	100 mg CoQ10, Multivitamin (Apovit), 1 g Omega-3 for 3 months	↓ DFI, no significant effect was seen on sperm concentration and on total motile sperm count
Knudson et al. 2021 [29]	2015–2019	RCT	67	65	Vitamin E, Selenium Zinc for up to 6 months	No correlation with semen parameters or clinical outcomes in couples with male infertility
Haidari et al. 2021 [24]	2014	Triple-Blind RCT	23	21	600 mg Lipoic acid or placebo for 3 months	↑ testosterone, ↑ GST gene expression, ↑ total sperm count, ↑ sperm concentration, ↑ sperm motility, no statistically significant changes in hormonal effects
Alahmar et al. 2020 [30]	2018–2019	Prospective RCT	33	33 (active control)	200 mg CoQ10, 200 μg selenium as an active control for 3 months	↑ sperm concentration, ↑ progressive and ↑ total motility but higher with Coq10, TAC, SOD, CAT (catalase), not significant change in selenium group, in sperm concentration and in sperm morphology
Aghajani et al. 2021 [31]	2018–2019	Parallel RCT	30	30 (active control)	7.5 ml carob syrup 100 mg, Vit E for 3 months	Superior in carob group the sperm count, motility and morphology, ↑ TAC and MDA
Eslamian et al. 2020 [32]	2013–2015	DB-RCT	124	40	465 mg DHA 600IU, Vit E or placebo for 3 months	↑ total sperm count, ↑ sperm concentration, ↑ sperm motility, ↑ progressive motility in DE group, not significant changes in morphology, ↓ 8-isoprostane ↑ TAC, ↓MDA
Nouri et al. 2019 [33]	2018	RCT	17	19	25 mg lycopene or placebo for 3 months	↑ total sperm count, ↑ sperm concentration, ↑ sperm motility
Stenqvist et al. 2018 [34]	2015–2016	DB-RCT	37	40	Antioxidant treatment (vitamins, antioxidants and oligoelements)	borderline statistically significant higher concentration, no change in DNA fragmentation index (DFI)
Alsalman et al. 2017 [35]	2011–2012	RCT	60	60	440 mg zinc sulfate or placebo for 3 months	Restored the oxidoreductive index and thiol-related enzyme activities
Alizadeh et al. 2017 [36]	2015–2016	DB-RCT	28	28	80 mg curcumin nanomicelle or placebo for 10 weeks	↑ total sperm count, ↑ sperm concentration, ↑ sperm motility. Statistically increased plasma levels of total antioxidant capacity, malondialdehyde, C-reactive protein, and tumor necrosis factor
Haghighian et al. 2015 [37]	2014	DB-RCT	23	21	alpha-lipoicacid (ALA) 600 mg or placebo for 12 weeks.	↑ sperm concentration, ↑ sperm count, ↑ sperm total motility

## Data Availability

Data is contained within the article.

## Supplementary Materials

The following supporting information can be downloaded at: https://www.mdpi.com/article/10.3390/jcm15020497/s1, Table S1: PRISMA checklist for scoping reviews; Table S2: Revised Cochrane Risk of bias/quality assessment tool for randomized trials.

## Abbreviations

The following abbreviations are used in this manuscript:

ALA	Alpha-Lipoic Acid
ATP	Adenosine Triphosphate
CI	Confidence Interval
CoQ10	Coenzyme Q10
DB-RCT	Double-Blind Randomized Controlled trial
DBPC	Double Blind Placebo-Controlled Trial
DFI	DNA fragmentation index
DHA	Docosahexaenoic acid
DNA	Deoxyribonucleic acid
FSH	Follicle-stimulating hormone
GST	Glutathione S-transpherase
ICSI	Intracytoplasmic sperm injection
IUI	Intrauterine insemination
IVF	In vitro fertilization
LA	Lipoic acid
LH	Luteinizing hormone
MDA	Malondialdehyde
MESH	Medical Subject Headings
OS	Oxidative stress
PRL	Prolactin
RCT	Randomized Controlled Trial
ROS	Reactive oxygen species
TAC	Total antioxidant capacity
VD3	Vitamin D3
WHO	World Health Organization
